# Female‐Specific Molecular Dysregulation in Alzheimer's Disease: miRNA and Gene Dynamics in 3xTg‐AD Mice

**DOI:** 10.1002/alz70855_100545

**Published:** 2025-12-23

**Authors:** Printha Wijesinghe, Jeanne Xi, Matthew Campbell, Si Xuan Chen, Wellington Pham, Joanne A Matsubara

**Affiliations:** ^1^ The University of British Columbia, Vancouver, BC, Canada; ^2^ Vanderbilt University Medical Center, Nashville, TN, USA; ^3^ Djavad Mowafaghian Centre for Brain Health, Vancouver, BC, Canada

## Abstract

**Background:**

Women are disproportionately affected by Alzheimer's disease (AD), accounting for approximately two‐thirds of cases. This disparity arises from multifactorial factors, including greater longevity, hormonal changes, genetic and biological differences, cognitive reserve, lifestyle, and sex‐specific pathophysiology. This study aims to investigate sex‐specific differences in microRNAs (miRNAs)—key post‐transcriptional and translational regulators of gene expression—and their associated roles in AD pathogenesis.

**Method:**

Using 3xTg‐AD mice, which exhibit cognitive impairments at 3–4 months of age and carry three familial AD‐associated mutations, along with their sibling controls, we analyzed animals of both sexes at 3, 6, and 12 months of age (4–6 animals per group). Fourteen miRNAs implicated in amyloidogenic, tauopathic, and inflammatory processes were screened in the neocortex‐hippocampus using TaqMan Advanced single‐tube miRNA assays. Additionally, 32 genes associated with amyloidogenic, tauopathic, and inflammatory pathways were analyzed in pooled neocortex‐hippocampus via RT‐qPCR for each age group, sex, and model. Relative expression levels were calculated as fold changes (>1.5‐fold) using the comparative Ct method, with statistical significance (*p* < 0.05) determined by three‐way ANOVA followed by Tukey's multiple comparison test.

**Result:**

Our analysis revealed significant miRNA dysregulation in female 3xTg‐AD mice at 3 months of age (equivalent to humans in their 20s). Notably, amyloidogenic miRNAs (‐15a, ‐29c, ‐101a, and ‐106b) were significantly downregulated in the neocortex‐hippocampus. Among the 32 genes tested, 27 showed differential expression, with 17 genes exhibiting significant downregulation. However, transgene APP expression was significantly and consistently upregulated across all time points (3, 6, and 12 months), while transgene MAPT expression was significantly upregulated specifically at 3 months in females. Inflammatory cytokines (Il6, Tnf, and Lif) were significantly upregulated, whereas neuroinflammatory markers (Gfap, Aif1, and Trem2) were markedly downregulated in the neocortex‐hippocampus. Additionally, genes related to APP clearance and microglial phenotypes, including Aqp4, Apoe, Abca1, Cfh, Clu, Cd68, Cx3cr1, P2ry12, Nfkb1, Stat3, and Sirt1, were also significantly downregulated.

**Conclusion:**

This study highlighted the vulnerability of female 3xTg‐AD mice at 3 months of age, marked by amyloidogenic miRNA dysregulation and differential gene expression in the neocortex‐hippocampus. However, sex‐specific differences in miRNA and gene expression diminished with aging.

